# Environment in Veterinary Education

**DOI:** 10.3390/vetsci10020146

**Published:** 2023-02-10

**Authors:** María del Pino Palacios-Díaz, Vanessa Mendoza-Grimón

**Affiliations:** Instituto de Estudios Ambientales y Recursos Naturales, Universidad de Las Palmas de Gran Canaria, 35017 Las Palmas, Spain

**Keywords:** environment, climate change, veterinarian education, one health, soil food web, GHG emissions, climate change

## Abstract

**Simple Summary:**

Environmental education is an important pillar for responding and adapting to climate change. The EU’s common agricultural policy (CAP) has evolved from rules which supported the farming sector after years of famine and has become oriented towards looking at environmental aspects. The CAP policies oriented towards optimizing natural resource use, residue management, antimicrobial use reduction, the decrease in greenhouse gas (GHG) emissions, and animal welfare, need educational programs linked to the environmental problems. In this context, veterinarians are experts in animal production, welfare, food safety, and its technology and in public health under the One Health concept. Unfortunately, they are barely trained in environmental aspects, which would help them to understand and face the consequences of climate change in the rural world. Veterinarians must be able to quantify the effects of animal production in the environment by using different analysis tools, which need to be included in their learning programs. In addition, they must be able to optimize the use of natural resources, minimize GHG emissions, and manage the risks associated with climate change.

**Abstract:**

Environmental concerns have become priority issues over the last third of the 20th century. The EU’s common agricultural policy (CAP) has gone from rules which supported the farming sector after years of famine to being oriented towards looking at environmental aspects. Therefore, it has evolved not only to react to a changing market and consumer demands but also to respond to climate change and the need for sustainable development. Environmental education is an important pillar for responding and adapting to climate change. The CAP policies oriented towards optimizing the use of natural resources, residue management, antimicrobial use reduction, the decrease of greenhouse gas emissions (GHG), and animal welfare need linked educational programs. In this context, veterinarians, being experts in animal production, welfare, and food safety and its technology and public health under the One Health concept, are scarcely informed in environmental aspects, which would help them to understand and face the consequences of climate change in the rural world. Future veterinarians must be able to quantify the effects of animal production on the environment, optimizing the use of natural resources, minimizing GHG emissions, and managing the risks associated with climate change by using different analysis tools that need to be included in their learning programs.

## 1. Introduction

Environmental concern has become a priority issue over the last third of the 20th century. The policy’s original goals are included in the Treaty of Rome. Launched in 1962, the EU’s common agricultural policy (CAP) supports farmers and ensures Europe’s food security [1]. While the CAP originally introduced rules for member states which would support the farming sector after years of devastating war and famine [2], it has progressively been re-oriented to look at environmental aspects. Therefore, it has evolved not only to react to a changing market and consumer demands but also to respond to climate change, biodiversity loss, and the need for sustainable development. Animals will feel the impact of climate change through multiple, often interacting, means, including changing patterns of infectious diseases, increased exposure to heat, contaminants, and extreme weather, changes in access to the natural resources they need for daily living, and shifts in animal ecology, sociobiology, and population dynamics [3].

The new concept of animal production has created environmental problems in certain geographical locations, which has led many countries to review their regulations with regard to intensive livestock farming and its inclusion in such regulations. This review aims to limit the direct effect of the livestock facilities themselves on the environment; it also considers the regulation of waste removal from livestock farms, with adequate management technologies to dispose of all the generated waste in an environmentally friendly manner. In Europe there is a payment for practices beneficial to the climate and the environment; this is also known as the “green payment”. This allows an annual payment to be granted for each admissible hectare linked to a basic payment right, if certain environmental practices are respected, and depending on the structure of the farm. Therefore, veterinarians need to know how to advise the farmers on how to proceed in exercising this right.

In this context, veterinarians who conduct their activities in rural zones must deeply understand these environment-related concepts. However, it is not clear whether veterinarian teaching programs are touching on these subjects, nor is it clear whether the veterinarians’ investigation aims are related to the discussed environmental issues. Hence, the purpose of this study was to determine: (i) what the main environmental issues are that veterinarians must know, particularly those working in the European context; (ii) whether there is enough content regarding the mentioned issues on the veterinary students’ syllabus, which is crucial to achieving sustainable rural development; and (iii) whether there are veterinarian publications related to environmental aspects; these are needed when proper education is encouraged.

## 2. Materials and Methods

To develop and justify the issues that veterinarians must know, we made a scientific (taking into account articles and projects) and normative review, including some tools useful to managing some of the concepts related to climate change. In addition, a study of the skills described by the Spanish Government to develop a veterinarian degree related to environmental issues was incorporated.

To demonstrate whether the above-mentioned issues were taught in veterinary schools, we presented (i) a scientific review; (ii) the results of an anonymous survey made of veterinarians from the USA and Canada to identify any educational gaps regarding veterinary medicine and climate change; (iii) the ideas exposed on some websites regarding veterinary education and veterinarian practices and climate change; and (iv) the results from a study of subjects related to the environmental issues of nine veterinary faculties in Europe: Alfort (France), Bologna (Italy), Budapest (Hungary), Hannover (Germany), Liège (Belgium), Liverpool (England, UK), Nantes (France), Uppsala (Sweden), and Utrecht (Netherlands). The original study included all the subjects taught in the selected veterinarian schools. As a criterion, at least one representative faculty per country was included; the faculty of Budapest was included as being representative of a European country not yet belonging to the EU, although of imminent entry; in the case of France, two centers (Alfort and Nantes) with recent changes in the structure of their degrees and different levels of application were included. Finally (v), a survey was carried out by the authors among the teachers from the Veterinary Medicine Faculty of the University of Las Palmas de Gran Canaria (ULPGC), asking whether environmental issues, climate change and the One Health (OH) concept were explicitly included in their teaching program and about the time spent during classes on these topics.

Finally, to describe the veterinary scientific publications related to environmental issues: (i) the scientific communications from the last (7th) OH congress related to these topics were analyzed (this congress was selected because OH is the environmental concept most related to veterinarian practices), and (ii) a mini-review was performed to describe the publications related to environmental issues and veterinarians. For this review, we initially evaluated the search strategy by considering the public databases of the peer-reviewed literature included in Academic Search Complete and PubMed from 2000 to the present, using keyword algorithms. Additionally, the same was performed from 2012 to the present, calculating the contribution from the last ten years and expressing it as a percentage of the published total. The following combinations of words were used: veterinary medicine + microbial + reduction or decrease; environment + veterinary; veterinary medicine + residue + environment; veterinary medicine + residue + water; antibiotics/antibacterial/antimicrobials + residue + environment; veterinary medicine + one health + environment; animal production + one health + environment; animal production + GHG (greenhouse gas); veterinary medicine + GHG; animal production + animal welfare + climate change; and veterinary medicine + animal welfare + climate change.

## 3. Results and Discussion

### 3.1. Environmental Issues That Veterinarians Must Know

One of the veterinarian practices is to advise the farmers on how to manage their livestock and residues and in the twenty-first century; sustainability and technology are critical factors in relation to these activities; so, they are going to be analyzed in this section. In the European context, the CAP indicates the activities which can be subsidized, and European farmers can rely on a more stable income through the CAP direct payments, weathering the impact of fluctuating prices and demand. Therefore, all the concepts included in the CAP must be known by European veterinarians. In this sense, special attention must be paid to GHG emissions and the use of technological tools. Since the early 2000s, farmers have received payments according to the area that they farm and not its output. Moreover, to protect the environment and safeguard Earth’s resources, direct payments remunerate environmentally friendly farming and public services which are not normally paid for by the markets, such as taking care of the countryside, preserving landscapes, protecting biodiversity, and helping to mitigate the impacts of climate change [2]. Thanks to the CAP, the European agricultural sector maintains some of the world’s highest safety and environmental standards. EU farmers are forced to practice environmentally sustainable farming. Therefore, they have to produce food whilst simultaneously protecting nature and safeguarding biodiversity. In this context, optimizing the use of natural resources to adaptively allocate them is essential for food production, guaranteeing the quality of life for current and future generations.

Once the new CAP comes into effect, we should see an increased contribution of EU agriculture to the environment and climate with the establishing of new schemes (so-called ‘eco-schemes’). The Green Deal objectives are extremely ambitious for EU farms and food systems, setting quantitative reduction targets by 2030 for pesticides, fertilizers, and antibiotics and aiming at a quantitative increase in organic farming, agricultural areas with high-diversity landscape features, and protected areas, focusing on the three related issues of climate, environment (notably biodiversity), and health [4]. The new CAP seeks to enhance the contribution of agriculture to the EU environmental and climate goals, to provide more targeted support to smaller farms, and to allow greater flexibility for member states in adapting measures to local conditions [5]. Fahad et al. [6] concluded that heat and drought are the two most important abiotic stresses that are exacerbated by climate change, with enormous impact on the growth and productivity of the crops. Consequently, animal productions are also affected by climate change; so, veterinarians need to properly understand and manage these changes. Conversely, climate change in temperate zones increases the vegetation period. Therefore, changes in agricultural systems and practices are needed to take advantage of the production growth during the more prolonged vegetative periods in temperate climates [7]. Rather than focus on risk management only, the programs will need to include capacity building for healthy, resilient animal populations and animal health systems [3]. In addition, the budget for the modernization of farming, which has grown consistently over the years in developed countries, is also used to help connect farmers with researchers and universities to fully unlock knowledge sharing. This partnership between agriculture and society has its complement in EU research priorities. Inter-professional education is needed to dissolve the siloes that have historically discouraged collaboration among professionals with different degrees and to increase the ability to solve the problems that frequently arise in rural zones.

The green practices which farmers can adopt include crop rotation or diversification and the maintenance of ecologically rich landscapes with a minimum amount of permanent grassland, as well as organic farming and carbon farming. The European Union countries are key producers and exporters of various agricultural products, such as pork, poultry, honey, milk, and eggs. Several of these agricultural products are covered by the Single Common Market Organisation, which provides one market [5]. The CAP policies oriented towards (i) antimicrobial use reduction, (ii) GHG emissions decrease (one of the main climate change goals), and (iii) animal welfare need educational programs. In addition, the role of ruminant livestock, the main extensive livestock system, and food security, with a particular emphasis on animal source foods and environmental sanitation, need to be taught in veterinarian programs.

Livestock models, which are mainly developed in Western Europe, lack the specific sustainability themes, which hinders the further rural development [8]. The authors highlight that model development would benefit from networks and multidisciplinary collaboration. In this sense, new tools based on the integration of geographical information systems (GIS) and multi-criteria are proposed to assess the risk of livestock farms, considering sectorial, social, and environmental criteria [9]. As an example, this overview is necessary to improve the surveillance systems, which are now based on compulsory notification to health authorities and are rather fragmented and limited with regard to the most severe diseases and only marginally consider the non-scientific community, while the initiatives characterized by trans-disciplinary collaboration may be more effective for the surveillance and prevention of transmitted diseases [10].

Specifically related to climate change and GHG emissions, the EX-Ante Carbon-balance Tool (EX-ACT) [11] has been developed by the FAO based on the Intergovernmental Panel on Climate Change (IPCC) methodology for GHG emission inventories by rural activities. This web tool (currently available in its v. 9.3.4) provides its users with a consistent way to estimate and track the outcomes of the agricultural interventions regarding GHG emissions. EX-ACT is the only GHG accounting tool to cover the entire agricultural sector, including agriculture, forestry, and other land use (AFOLU,) inland and coastal wetlands, fisheries and aquaculture, agricultural inputs, and infrastructure. Recently, an extension tool has been developed to account for the value chains (EX-ACT VC) [12]. At the EU level, there are also web tools related to environmental aspects and farming, such as the AGRIADAPT tool [13]. The objective of the AGRIADAPT project, developed under the LIFE program of the European Commission, was twofold: to assess the vulnerability of the main European agricultural products to climate change and to propose sustainable adaptation plans allowing these systems to become more resilient. The tool was based on the monitoring results of more than 120 pilot farms located in France, Spain, Germany, and Estonia, aiming to strengthen the agroclimatic knowledge of the users and to support them in moving towards a more adapted agriculture. Additionally, and as an example, the Spanish Government has recently established an ECOGAN web tool, as farm owners are responsible for estimating their emissions and applying the best available techniques (BATs), to avoid or, when this is not possible, reduce emissions and the impact on the whole environment. These tools will improve future veterinarians’ skills.

To address the effect of climate change on health in a broader context, some authors have described the link between humans, animals, and their environmental and social contexts [14,15]. The conceptual framework of One Health (OH) provides a strategy for the promotion of collaboration across the nexus of animal, human, and environmental health, which is essential for tackling emerging disease threats [16]. Rweyemamu et al. [17] concluded that the lessons learnt from recent zoonotic epidemics clearly indicate the need for coordinated research, interdisciplinary centers, response systems and infrastructures, integrated surveillance systems, and workforce development strategies. The authors pointed out the importance of initiatives such as “The SACIDS”, a One Health African initiative linking southern African academic and research institutions with international research institutions; it strives to strengthen Africa’s capacity to better manage the risks posed by infectious diseases and to improve the research capacity in investigating the biologic, socio-economic, ecologic, and anthropogenic factors responsible for the emergence and re-emergence of infectious diseases. A successful “One Health” strategy was implemented in Mongolia under the concept of “Healthy Animal-Healthy Food-Healthy People” [18], integrating not only the veterinary and public health sectors but also incorporating more work on food safety, emergency management, and the effects of climate change on zoonotic diseases. As concluded by Zinsstag et al. [19], “A One Health approach to climate change adaptation may significantly contribute to food security with emphasis on animal source foods, extensive livestock systems, particularly ruminant livestock, environmental sanitation, and steps towards regional and global integrated syndromic surveillance and response systems”.

The soil food web (SFW) is an essential component of a healthy ecosystem, as it naturally supports plant health by providing protection against pests and diseases and provides fundamental ecosystem goods and services. The spread of antibiotic resistance genes (ARGs) represents a major threat to public health in which the SFW can play an important role by providing an additional treatment against this contamination. For instance, Han et al. (2022) [20] studied the field application of organic fertilizers (the long-term fertilization of swine manure or sewage sludge), which may promote the spread of ARGs in farmland ecosystems. The plant phyllosphere is a tough habitat for microbes with limited nutrients, high radiation, and frequent alteration of relative humidity, temperature, and wind speed. Their results demonstrated that the long-term application of swine manure and sewage sludge differently impacts the ARGs in soil and the phyllosphere, which has implications for sustainable agricultural management.

The American Veterinary Medical Association (AVMA) asserts that veterinary expertise in toxicology, epidemiology, and ecology are vital to understanding, controlling, preventing, diagnosing, and treating the environment-associated diseases that affect both people and animals. Although these concepts, as well as animal production, are included in all of the veterinarian students’ programs, there is a lack of global vision and a more holistic approach should be included to properly include environmental issues. The most actualized document defined by the Spanish Government, called “*Libro Blanco de Veterinaria*“ [21], which is to evaluate Spanish veterinarian studies and in which the main disciplinary competences to be known by the students are established, describes the following skills regarding environmental issues (Table 1).

Therefore, veterinarians must be able to quantify the effects of animal production in the environment using different tools, which must be included in their learning programs. Furthermore, they must be able to optimize the use of natural resources, in order to minimize GHG emissions and to manage the risks related to climate change through different strategies, including the ones derived by One Health knowledge.

### 3.2. Is Enough Knowledge Taught in Veterinary Schools to Familiarize Their Students with Environmental Issues?

Once we determine the main environmental issues that veterinarians must know, a question arises with regard to their coverage in existing teaching programs. Roopnarine et al. [16] pointed out that there is no accreditation requirement for One Health to prepare students across the professions for collaborative practice. Environmental studies seem to be barely developed in veterinarian programs, although veterinarians usually collaborate with other professionals related to environmental studies. Although the soil food web is an essential component of a healthy ecosystem, soil is scarcely included in the veterinarian view of One Health. An anonymous survey was made of veterinarians from the USA and Canada to identify any educational gaps regarding veterinary medicine and climate change. Although the veterinarians agreed that the profession needs to be involved with climate change advocacy, most reported having had no educational opportunities within their veterinary medicine curriculum or access to continuing education on climate change. The study concluded that there is a need to develop educational programs on the topic of climate change such that veterinarians are equipped to address their concerns about current and future animal health threats [22]. In this sense, some websites, such as that of the Michigan State University College of Veterinary Medicine, raise awareness about climate change by explaining the role of veterinary medicine and encouraging their students to sign up for the Environmental Wellness Committee. In addition, the Global Consortium on Climate and Health Education [23] was created by Columbia Public Health. Moreover, the American Veterinary Medical Association (AVMA) [24] encourages research and education to improve the understanding of the impacts of climate change on animal, human, and ecosystem health on its website, under the “Global Climate Change and One Health” tab.

The Association for Prevention Teaching and Research (APTR) [25] invited the American Association of Veterinary Medical Colleges (AAVMC) [26] to develop a One Health educational framework as a part of the Healthy People Curriculum Task Force (HPCTF). A working group comprising representatives from the AAVMC, the APTR, the American Association of Colleges of Nursing, the Association of American Medical Colleges, and other groups has established a program to develop case studies in inter-professional education and to nominate One Health scholars. In addition, Rabinowitz et al. [27] concluded that, while many medical educators may not yet be familiar with the concept, the One Health approach has been endorsed by several major medical and public health organizations and that it is beginning to be implemented in several medical schools. As an example, a massive open access online course: “One Health: Connecting Humans, Animals and the Environment” [28] has 10,523 enrolled students. In addition, many open access documents of OH resources for public health educators are available in the One Health Commission web page [29]. Finally, there are some articles requesting the expansion of education for veterinary professionals and students in policy and advocacy; calls to action are established to address climate change and planetary health issues [30].

In the last study carried out for the Government of Spain on the curricula of veterinary schools in Europe [21], a total of nine centers were analyzed, including the basic structure of the subjects developed by their programs. There was no subject which mentioned climate change or One Health in either of the faculties, while the subjects related to environmental issues are described in Table 2.

The above-mentioned document [21] also includes the labor insertion of university graduates, and only 0.69% of the veterinarians worked in environmental activities, while 2.08% of them did it as a secondary activity. Of the asked graduates, 0.7% had already had their first work related to these activities.

**Figure 1:**
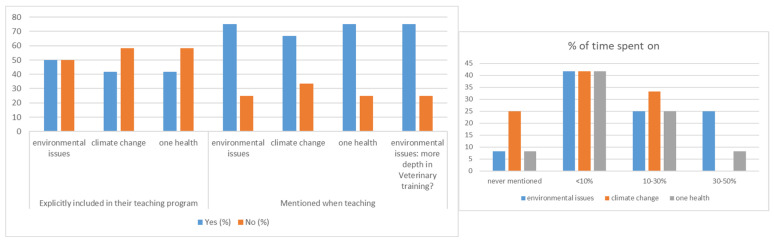
Environmental issues, climate change, and One Health (**left**) and time (%) spent on teaching these issues (**right**), in veterinary studies in Veterinary Medicine Faculty ULPGC.

Despite the fact that environmental issues are not structurally included in veterinarian studies, many of the factors are often taught as stand-alone factors. These factors link medical and public health issues to climate change, such as water-related health issues, the high frequency of extreme weather events, the increasing global temperature, changes in vector-borne diseases, and scarce resources related to food safety/security. As an example, in a survey carried out by these authors among the teachers from the Veterinary Medicine Faculty of the University of Las Palmas de Gran Canaria (ULPGC), 50% of them stated that environmental issues were explicitly included in their teaching program, while 75% incorporated environmental aspects during classes, adding up to more than 10% of the total time being taken by the 50% of the teachers (with only one teacher’s answer being that environmental notions were never mentioned). A slightly lower percentage (42%) of the teachers refer specifically to climate change in their teaching programs and, although 67% give some ideas related to this concept, with more than 10% of the teaching time being taken by 33% of the teachers, 25% of them never mention climate change during their lectures (Figure 1). Finally, One Health obtained the same answers as the environmental issues; so, it can be argued that OH is the main environment-related concept perceived by the veterinarian teachers. As concluded in a free webinar to prepare health professionals to meet the needs and challenges of the future, educators worldwide are called upon to integrate climate change studies into health curricula [31].

There is less information on the challenges and training needs of veterinarians in sustainable development and animal production in the face of climate change. However, veterinarians must also be supported and formally trained in animal production and public policies in the face of climate change if they want to maintain their commitment to promoting public health and sustainable development. The new European regulatory requirements incorporate territorial livestock planning as a key element to correct the risks for the sector, for the nearby population, and for the environment. Therefore, GIS and the multi-criteria tools described above should be included in veterinarian learning programs, to allow graduated students that have acquired the new skills to be able to work as private advisers or to allow them to better integrate in multidisciplinary working groups.

The veterinarians of the future must have a vision beyond animal health and production and need to understand the links between the different subjects studied. Innovative learning methodologies are very well suited to the introduction of environmental issues in veterinary education. As pointed out by Dooley and Bamford [32], collaborative learning activities in the context of veterinary education can provide an opportunity to scaffold the development of crucial core competencies, including the self-regulated learning skills required to work in collaborative teams and to interpret and act on feedback.

### 3.3. Veterinarian Scientific Publications Related to Environmental Aspects

Regarding the last aim, veterinarian publications related to the environment, different sources have been consulted: the last (7th) OH congress [33] and two scientific databases: Academic Search Complete and PubMed. In this sense, in the “One Health Science Session of oral presentations and posters” from the 7th One Health congress, more than 70% of communications were strictly linked to environmental aspects. In this congress, analyzing specifically the use of veterinary antimicrobial pharmaceuticals and their residue reduction, 11% of the oral presentations and posters were related to antimicrobial resistance. Finally, 16% of the oral presentations and posters were related to policy, environment and biosecurity. As an example, Magiri et al. [34] describe the fact that there is a considerable overlap in the antibiotic classes sold for use in both human and veterinary medicine, mainly the beta lactam/penicillin, tetracycline, sulfonamide, and macrolide antibiotic classes.

The results of the mini-review, conducted to describe the publications related to environmental issues and veterinarians are presented in Table 3. The combination of words which presents the highest number of publications is: animal production + one health + environment, presenting more than double that of any other combination, showing the deep relation of OH with veterinary practices. The second combination differs between the databases considered: antibiotics/antibacterial/antimicrobials + residue + environment (Academic Search Complete) and environment + veterinary (PubMed). The combination of words which presented the lowest number of publications for both of these databases included GHG plus animal production or veterinary medicine. However, these combinations presented the highest percentage of publications in the last ten years (in a range from 86% to 89% in Academic Search Complete and 95% to 99% in PubMed), showing the increasing importance of this topic nowadays.

## 4. Conclusions

While veterinary medicine, which plays a critical role in protecting the public from zoonotic infectious diseases, the safety of the food supply, and biomedical research are gaining importance within One Health, the education of veterinarians is currently less linked to the animal production and welfare related to climate change. Expanding their education would help veterinarians understand and face the consequences of climate change in the rural world. Deep changes must come to prepare the students to reduce the effects of animal production on the environment, using different tools which must be included in the learning programs. In addition, they must be able to optimize the usage of natural resources, to minimize GHG emissions, and to manage risks through different strategies. Collaborative learning activities can provide an opportunity to scaffold the development of crucial core competencies, including the self-regulated learning skills required to work in collaborative environmental teams.

## Figures and Tables

**Table 1:** Skills described by Spanish Government to develop veterinarian degree and its relation to environmental issues.

Skills		Mentioned	Related
Discipline	6. Know the basics of the different biological agents of veterinary interest (veterinary medicine/animal production and health)		X
	8. Knowledge and diagnosis of the different animal diseases, individual and collective, and their prevention measures, with special emphasis on zoonoses and notifiable diseases (all)		X
	10. Knowledge of the bases of the operation and optimization of animal production systems and their repercussions on the environment (animal production and health)	X	
Professional	5. Identify, control, and eradicate animal diseases, with special attention to notifiable diseases and zoonoses (animal production and health/veterinary medicine)		X
	9. Advise and carry out epidemiological studies and therapeutic and preventive programs in accordance with the standards of animal welfare, animal health and public health (animal production and health)		X
	11. Manage specific protocols and technologies aimed at modifying and optimizing the different animal production systems (animal production and health)		X
	14.Carry out risk analysis, including environmental and biosafety risks, as well as their assessment and management (hygiene, safety and food technology/animal production and health/others)	X	
	16. Advice and management, technical and economic, of companies in the veterinary field in a context of sustainability (all)		X

**Table 2:** Subjects related to environmental issues of nine Veterinary Faculties in Europe.

University	Subject	Mandatory	Optional	Clinical Studies
Alfort	Environmental Toxicology			X
Bolonia	-	-	-	-
Liverpool	Animals and Environment	X		
Uppsala	-	-	-	-
Lieja	-	-	-	-
Budapest	-	-	-	-
Hannover	-	-	-	-
Nantes	Environment and Toxicological Clinic	X		
	Environmental Toxicology		X	
Utrecht	Environment	X		

**Table 3:** Results from the mini-review conducted to quantify the number of scientific articles related to environmental issues and veterinarians published in 2000 to 2022 period and them percentage of them published during the last ten years.

	Academic Search Complete		PubMed	
	2000–2022	% (2012–2022)	2000–2022	% (2012–2022)
veterinary medicine + microbial + reduction or decrease	5269	74	3321	84
environment + veterinary	16,465	77	12,380	87
veterinary medicine + residue + environment	4530	70	2176	84
veterinary medicine + residue + water	4800	72	2307	86
antibiotics/antibacterial/antimicrobials + residue + environment	19,570	79	8126	89
veterinary medicine + one health + environment	13,111	74	9003	86
animal production + one health+ environment	56,113	77	35,108	90
animal production + GHG	1081	89	536	99
veterinary medicine + GHG	99	86	59 *	95
animal production + animal welfare + climate change	4674	77	1969	88
veterinary medicine + animal welfare + climate change	1230	75	801	89

* there were no publications before 2010.

## Data Availability

The data presented in this study are available upon request from the corresponding author.
